# A randomized controlled clinical trial of the effect of supportive counseling on mental health in Iranian mothers of premature infants

**DOI:** 10.1186/s12884-020-03502-w

**Published:** 2021-01-05

**Authors:** Leila Seiiedi-Biarag, Mojgan Mirghafourvand, Khalil Esmaeilpour, Shirin Hasanpour

**Affiliations:** 1 Department of Midwifery, School of Nursing and Midwifery, Tabriz University of Medical Sciences, Tabriz, Iran; 2 Social Determinants of Health Research Center, Faculty of Nursing and Midwifery, Tabriz University of Medical Sciences, Tabriz, Iran; 3 Faculty of Education and Psychology, University of Tabriz, Tabriz, Iran; 4 Women’s Reproductive Health Research Center, Nursing and Midwifery Faculty, Tabriz University of Medical Sciences, Tabriz, Iran

**Keywords:** Premature, NICU, Mothers mental health, Supportive counseling

## Abstract

**Background:**

Premature birth can affect maternal mental health. Considering that the mental health disorder in mothers may play a vital role in the growth and development of their children, therefore, this study was conducted to determine the effect of supportive counseling on mental health (primary outcome), mother-child bonding and infant anthropometric indices (secondary outcomes) in mothers of premature infants.

**Methods:**

This randomized controlled clinical trial was carried out on 66 mothers with hospitalized neonates in the NICU of Alzahra hospital in Tabriz- Iran. Participants were randomly allocated into two groups of intervention (*n* = 34) and control (*n* = 32) through a block randomization method. The intervention group received 6 sessions of supportive counseling (45–60 minutes each session) by the researcher, and the control group received routine care. Questionnaires of Goldberg General Health and the postpartum bonding were completed before the intervention (first 72 hours postpartum) and 8 weeks postpartum. Also, the anthropometric index of newborns were measured at the same time.

**Results:**

There was no statistically significant difference between the two groups in terms of socio-demographic characteristics. After the intervention, based on ANCOVA with adjusting the baseline score, mean score of mental health (AMD: -9.8; 95% Confident Interval (95% CI): -12.5 to -7.1; *P* < 0.001) and postpartum bonding (AMD: -10.0; 95% CI: -0.6 to 13.9; *P* < 0.001) in the counseling group was significantly lower than those of the control group; however, in terms of weight (*P* = 0.536), height (*P* = 0.429) and head circumference (*P* = 0.129), there was no significant difference between the two groups.

**Conclusions:**

Supportive counseling may improve mental health and postpartum bonding in mothers of premature infants. Thus, it may be recommendable for health care providers to offer it to mothers.

**Trial registration:**

Iranian Registry of Clinical Trials (IRCT): IRCT20120718010324N45. Date of registration: October 29, 2018.

## Background

Approximately, 15 million infants are born prematurely each year [1]. Of these, around 12% are born in low-income countries and 9% in high-income ones [2]. In Iran, according to a meta-analysis published in 2017, preterm birth rates have been reported 10% [3]. Premature infants are exposed to difficulties such as neurological, developmental [4], motor, behavioral [5], vision [6], and hearing impairments [7]. After the birth of premature infants and the hospitalization at NICU, the normal process of caring the infants and the parental role, particularly that of the mother, are highly affected. Therefore, the mother may be psychologically, physically, and emotionally be not ready to admit the experience [8]. Such mothers may experience various mental health issues such as depression, anxiety, and stress [9]. Parents’ concerns about the health and survival of their infants, loss of parental roles, health care providers [10], the appearance and behavior of infants, alarm bell noise, and other medical equipment in the NICU are significant stressors for these parents [11]. Mothers’ distressing memories of NICU may continue up to 6 months after childbirth and lead to traumatic stress disorder in them [12]. Parental mental health state plays a vital role in the cognitive, social [13], emotional, and behavioral development [14] of children. Mothers who have mental health issues spend little time with their children, thereby causing developmental disorder in their babies [15].

Furthermore, mothers’ mental health disorders, such as depression, may hurt infant nutrition and mother-child bonding [16]. The mother-child bonding is an emotional relationship between mother and baby that arises during pregnancy [17] and develops in the postpartum period through the mother and infant relations [18]. Mother-child bond formation raises mother self-confidence and her ability to provide care for the infant [19]. However, a disorder in postpartum bonding causes issues such as separation anxiety disorder, avoidant personality disorder, delinquency, and educational difficulties [20]. In parents of premature infants, parental expectations (as compared with postpartum experiences), maternal response, and psychological symptoms in mothers may have the most significant impact on postpartum bond formation. Also, the evidence indicates that mental health disorder in mothers is one of the causes of infant failure to thrive [21]. The results of a study showed that maternal mental problem during the prenatal and postpartum periods predicts poor child growth [22]. It seems essential, therefore, to support these mothers and to intervene to reduce their psychological symptoms and accordingly to facilitate the bond between mother and child [23] and improve child outcomes [21].

According to the findings of Shaw et al. (2013), psychological support should be offered to improve the parent relationship with their premature infant and to reduce the following long-term and severe consequences. The researchers have suggested that counseling and psychotherapy are effective interventions to support these families [24]. Emotional support by health care providers prevents mothers’ emotional problems and plays a vital role in helping mothers with depression or at risk for postpartum depression [25]. The results of related studies reveal that supportive counseling may be useful in preventing mental health disorders [26, 27]. In this type of counseling, which consists of factors such as empathy, listening, encouragement, explanation and training, reassurance, guidance, and practical help, the counselor encourages the individuals to talk about their issues such as guilt and anger and also teach them the mechanisms and strategies to restore the quality of life to the initial state [28].

Considering the increasing trend of preterm birth [2], its high resulting mortality and morbidity [29], and consequently the issues in maternal mental health and mother-child bonding [30], on one hand, lack of study in Iran, on the other hand, the present study aimed to evaluate the effect of supportive counseling on mental health in Iranian mothers of premature infants (primary outcome), mother-child bonding and infant anthropometric indices (secondary outcomes) in mothers of premature infants.

## Methods

### Study design and participants

This single-blind randomized controlled clinical trial with two parallel arms was carried out on 66 mothers with premature infant hospitalized in the neonatal intensive care unit of Alzahra hospital in Tabriz, Iran. Al-Zahra Hospital (a tertiary referral hospital located in Tabriz city), consists of two NICUs equipped with the most advanced devices, such as warmer, ventilator, phototherapy, incubator and several modern monitors. In this hospital, a total of 63 nurses (20 nurse aids, 2 midwifes, 2 secretaries, and 2 servants in NICU) offer the care services. Only the data analyzer was blinded to the intervention received by the study groups to communicate with the mother and the mother’s unwillingness to participate.

Inclusion criteria included: having at least secondary education, infants born between 28 and 33 weeks and being 6 days hospitalized in the NICU, having a contact phone number, residing in Tabriz, and having birth less than 72 hours. It has to be noted that to prevent information exchange between counseling and control groups, mothers residing in Tabriz who did not stay with other mothers in the mothers’ room in hospital, were selected as the participants. Exclusion criteria included: not being sure about attending all counseling sessions, taking psychiatric medication and antidepressants, history of pre-pregnancy psychiatric problems, existence of any congenital anomaly in the infant^’^s and mother hospitalization for more than 72 hours. Women ages lower than 16 were excluded from the study. Mothers who had infants under 28 weeks were excluded from the study due to higher likelihood of infant death and also mothers who had infants over 34 weeks were excluded from the study due to early discharge of infants from hospital that could be reduced mothers’ participation in the counseling sessions).

According to Ghafari et al. [31], using G-Power software with m_1_ = 9.52 and 15% decrease in average mental health score due to intervention (m_2_ = 8.09), sd_1_ = sd_2_ = 1.58, two-sided α = 0.05, Power = 90%, sample size was calculated to be 27 per group. Considering a 20% loss, the final sample size was 66, of which 34 were placed in the counseling group and 32 in the control group.

### Procedures

This study was initially approved by the Ethics Committee of Tabriz University of Medical Sciences with the code of IR.TBZMED.RES.1397.693. Then, after trial confirmation at the Iranian Registry of Clinical Trials (IRCT) with the code of IRCT20120718010324N45, the researcher started the sampling. Initially, the researcher attended in the NICU of Al-Zahra Hospital and extracted a list of infants who were born in less than 72 hours and invited their mothers to participate in the study. If mothers wish to participate in the study, they were provided with more information on the goals, the importance, benefits of participating in the study, and the stages of conducting the research. Next, they were examined to be sure whether to meet the needed criteria. When the mothers were found eligible, The GHQ was completed by the researcher through interview. Written informed consent was received from people with a total mental health score less than 24. Other research questionnaires including socio-demographic information and PBQ were completed by the researcher, as well. The researcher also inserted the infant height, weight, and head circumference (at birth) through the infant’s file into the anthropometric index checklist.

### Randomization

Allocation sequence was generated by a computerized random number generator. The participants were randomly assigned to two groups of intervention (supportive counseling) and control through block randomization method with block sizes of four and six and an allocation ratio of 1:1. Randomization was stratified by the gestational age (28 to 30 weeks and 6 days; 31 to 33 weeks and 6 days) and parity (nulliparity; multiparity). Blocking was performed by the non-involved person in data sampling and analysis. The intervention type was written on paper and placed in opaque envelopes, numbered consecutively to hide the allocation (Allocation Concealment).

### Intervention

For the intervention group, six 45–60 minutes session of supportive group counseling was performed by researcher in the teaching room of Alzahra Hospital. Each group was composed of four or five mothers. Accordingly, six counseling sessions were performed, the first session within the first 72 hours of birth and then at the end of the first week and thereafter twice a week until the third week. Also, in the first session, booklets containing the content of the consultation were presented to mothers in the intervention group. The materials offered to the intervention group were as follows:

#### First session

Introducing, welcoming, and setting a meeting agenda; explaining the purpose of the project; listening to clients and understanding their mental concerns; sympathizing with clients; explaining preterm birth, its causes, and the status of preterm infants; explaining the NICU; and answering to participant questions.

#### Second session

Determining the meeting agenda, reviewing the previous session, expressing the adverse effects of stress on mothers and their babies, teaching baby massage through video, and accordingly expressing its importance and answering to participant questions.

#### Third session

Determining the meeting agenda, reviewing the previous session, allowing mothers to talk about their feelings and concerns, encouraging clients’ positive thoughts and abilities, and answering to participant questions.

#### Fourth session

Determining the meeting agenda, reviewing the previous session, supporting mothers in expressing emotions, encouraging clients’ abilities, training Jacobson’s relaxation technique, providing opportunities for information exchange between mothers who received counseling, and answering to participant questions.

#### Fifth session

Determining the meeting agenda, reviewing the previous session, addressing mothers’ confusions, explaining risk symptoms in preterm infants, providing opportunities for information exchange between mothers who received counseling and answering to participant questions.

#### Sixth session

Determining the meeting agenda and reviewing the plan, supporting mothers’ emotions and answering their questions, explaining on premature infant care at home, providing opportunities for information exchange between mothers who received counseling explaining achievement retention, and continuously following education throughout the life course.

All counseling session was provided to the intervention group by the first author (LSB). She was trained about Jacobson’s relaxation by her supervisor (KE, Associate Professor of Psychology). For training of baby massage, an educational film was used. At the end of each session, recorded video of relaxation and massage training was given to mothers. Moreover, the researcher’s phone number was given to the intervention group in order to be in contact with the researcher in case they need guidance after leaving the hospital. The control group received routine care (breastfeeding training). Once again, the Mental Health Questionnaire, Postpartum Bonding Questionnaire and the anthropometric indices checklist (including height, weight, and head circumference of the infants) were completed at week 8 postpartum. After completing the follow-up, a summary of the contents of counseling sessions was provided to the control group.

### Measurements

The questionnaires of socio-demographic and obstetrics characteristics, GHQ, and postpartum bonding, as well as anthropometric index registration checklist of neonates, were used for data collection.

Socio-demographic and obstetrics characteristics questionnaire included questions about age, marital status, education level, employment status, monthly income adequacy (this item was assessed subjectively and the response options were “completely adequate”, “somewhat adequate” and “inadequate”), number of pregnancies, and pregnancy and childbirth history.

In this study, a 28-item General Health Questionnaire was used to assess mental health. GHQ was first designed and launched by Goldberg in 1972 [32]. The questionnaire consists of four sub-scales, each containing seven questions. The scales of this questionnaire are as follows: (1) physical symptoms, (2) anxiety and insomnia, (3) social dysfunction, and (4) depressive symptoms. The total score of each individual is obtained by summing the scores of these four scales. A low score on this scale is a sign of health and a high score is a sign of being unhealthy. The 4-point Likert scale (with scores of 0, 1, 2, and 3) is used for scoring. An overall score of 24 and above indicates higher levels of distress [33]. According to Nazifi et al. [34], this questionnairehas adequate validity and reliability for measuring mental health. The Cronbach’s alpha coefficient of all sub-scales of that was above 0.74. This result is in line with the findings of the Taghavi who had reported the Cronbach’s alpha coefficient more than 0.9. In the present study, the reliability of the mental health tool was determined by a test-retest (10 days interval) on 20 women and determining the ICC and internal consistency (Cronbach’s alpha coefficient). For this questionnaire, Cronbach’s alpha coefficient was 0.881 and ICC (95% Confidence Interval) was 0.887 (0.680 to 0.950).

The PBQ consisted of 25 items indicating the mother’s feelings and attitude toward her infant. Participants express their feelings on a 6-point Likert scale (0–5). Lower scores imply an excellent relationship. The PBQ has 4 sub-scales [35], with subscale 1 reflecting disorder in relationship (12 items; score 0–60), subscale 2 reflecting rejection and pathological anger (7 items; score 0–35), subscale 3 reflecting anxiety about caregiving (4 items; score 0–20), and the subscale 4 reflecting the risk abuse (2 items; score 0–10). The suggested cut-off points for identifying problematic relationships are as follows: 12 for subscale 1, 17 for subscale 2, 10 for subscale 3, and 3 for subscale 4. The cut-off point for the entire scale was considered to be 38. In the work of Aflak Seir and Jamali [36], content validity was obtained using the opinions of faculty members and its reliability using Cronbach’s alpha coefficient method of 0.52, 0.67, 0.70 and 0.74 for components of a defective relationship, rejectionand anger, caregiving anxiety, and the risk of abuse, respectively. In this study, through test-retest (10 days interval), Cronbach’s alpha coefficient of 0.862, and ICC (95% CI) of 0.829 (0.517 to 0.924) were calculated for this questionnaire on 20 subjects.

Anthropometric index registration checklist include questions about the infant’s age, height, weight, and head circumference that were recorded before the intervention from the infants’ files (at birth), and also were measured and recorded by the researcher 8 weeks after the intervention using scale and tape measures.

### Statistical analysis

Data were analyzed by SPSS version 21. The Kolmogorov-Smirnov test was used to determine the normality of the quantitative data. To compare neonatal anthropometric indices and the mean scores of postpartum bonding and maternal mental health between groups, independent t-test was used before intervention and ANOVA test adjusted for the baseline values and stratification factors (parity and gestational age), after the intervention. All analyses were performed based on intention-to-treat. *P* < 0.05 was considered as the significance level.

## Results

**Fig. 1:**
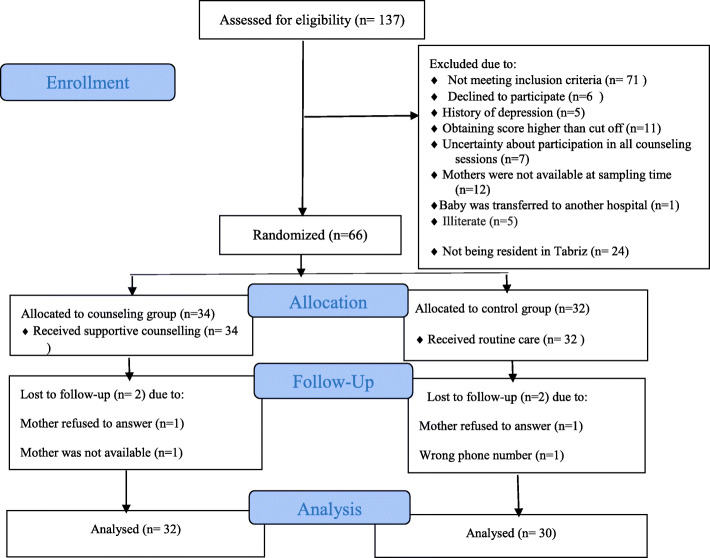
Flow chart of the study

In this study, which was performed from October 2018 to May 2019 at Al-Zahra Hospital in Tabriz, 137 mothers were examined for eligibility, of which 71 did not meet the inclusion criteria (Fig. 1).

The mean (SD) age of mothers was 28.2 (6.0) in the counseling group and 29.4 (7.2) in the control group. As regards employment, the majority of mothers in both groups (88% in the counseling group and 78% in the control group) were housewives with no significant difference between the two working hours. Approximately, 21% of mothers in the counseling group and 34% in the control group had a university education. Also, 21% of the participant’s husbands in the counseling group and 28% in the control group had a university degree. Regarding the husband job, about 32% of the spouses in the counseling group and 41% in the control group were workers. The majority of mothers in both groups (68% in the counseling group and 67% in the control group) stated that their monthly income is partially adequate for living expenses. Around four-quarters of mothers in both groups were completely satisfied with their family relationships (79% in the counseling group and 75% in the control group). Almost 18% of mothers in the counseling group and 3% in the control group had a history of infertility. About 32% of mothers in the counseling group and 31% in the control group had an abortion experience. The mean (SD) gestational age in the counseling group was 220.7 (12.3) days while in the control group it was 219.9 (13.8) days. In terms of parity, more than half of the mothers in both groups were nulliparous (62% in the counseling group and 59% in the control group). Preterm Premature rupture of membranes (PPROM) in 47% of the mothers in the counseling group and 37% in the control group had resulted in preterm birth.

Further, 44% of the mothers in the counseling group and 50% in the control group had premature birth for other reasons such as labor pain, hypertension, preeclampsia, and placental abruption. More than half of the infants in both groups were boys (60% in the counseling group and 56% in the control group). The majority of infants in both groups (94% in the counseling group and 96% in the control group) required artificial ventilation in first 72 hours postpartum. Moreover, about two-thirds of infants (in first 72 hours postpartum) in both groups needed an incubator (68% in the counseling group and 67% in the control group). Only 3% of infants in the control group had intrauterine growth restriction. Approximately, 27% of infants in the counseling group and 13% in the control group were small with respect to the pregnancy age. The mean (SD) hospitalization period of infants was 19.6 (13.1) days in the counseling group and 25.4 (17.6) days in the control group (Table 1).

**Table 1:** Socio-demographic and obstetrics characteristics of the participants

Characteristics	Counseling group *n* = 34 Number (Percent)	Control group *n* = 32 Number (Percent)
**Age** (year), Mean (SD)	28.2 (6.0)	29.4 (7.2)
**Husband’s Age** (year), Mean (SD)	33.8 (7.0)	33.9 (6.2)
**Job**		
House keeper	30 (88.2)	25 (78.1)
Employed	4 (11.8)	7 (21.9)
**Level of education**		
Secondary School	6 (17.6)	13 (40.6)
High School	3 (8.8)	1 (3.1)
Diploma	18 (52.9)	7 (21.9)
University	7 (20.6)	11 (34.4)
**Husband’s education**		
Illiterate	0	2 (6.3)
Primary School	4 (11.8)	5 (15.6)
Secondary School	6 (17.6)	2 (6.3)
High School	4 (11.8)	5 (15.6)
Diploma	13 (38.2)	9 (28.1)
University	7 (20.6)	9 (28.1)
**Husband’s Job**		
Unemployed	1 (2.9)	1 (3.1)
Worker	11 (32.4)	13 (40.6)
Employed	6 (17.6)	5 (15.6)
Shopkeeper	4 (11.8)	4 (12.5)
Others	12 (35.3)	9 (28.1)
**Monthly income adequacy**		
Completely adequate	5 (14.7)	3 (9.4)
Somewhat adequate	23 (67.6)	21 (65.6)
Inadequate	6 (17.6)	8 (25)
**Satisfaction of family relationship**		
Completely Satisfied	27 (79.4)	24 (75)
Somewhat Satisfied	6 (17.6)	6 (18.8)
Not satisfied –Not dissatisfied	1 (2.9)	2 (6.3)
Somewhat Dissatisfied	0	0
Completely Dissatisfied	0	0
**History of infertility**	6 (17.6)	1 (3.1)
**History of abortion**	11 (32.4)	10 (31.3)
**Gestational age (day),** Mean (SD)	220.7 (12.3)	219.9 (13.8)
**Number of parity**		
1	21 (61.8)	19 (59.4)
2	11 (32.4)	9 (28.1)
≥ 3	2 (5.8)	4 (12.5)
**Reason of preterm birth**		
Preterm Premature Rupture of Membranes	16 (47.1)	12 (37.1)
Placenta Previa	2 (5.9)	3 (9.4)
Placental Abruption	1 (2.9)	1 (3.1)
Others	15 (44.1)	16 (50)
**Infant’s sex**		
Female	15 (44.1)	14 (43.8)
Male	19 (55.9)	18 (56.3)
**Need for ventilation**	32 (94.1)	31 (96.2)
**Need for incubator**	23 (67.6)	21 (65.6)
**Intrauterine Growth Restriction**	0	1 (3.1)
**Small for Gestational Age**	9 (26.5)	4 (12.5)
**Length of hospital stay (day)**	19.6 (13.1)	25.4 (17.6)

The mean (SD) of the total mental health score before the intervention was 19.8 (2.4) in the counseling group and 18.6 (2.6) in the control group. There was a statistically significant difference between the two groups before intervention based on the independent t-test (*P* = 0.048). The mean (SD) of the total mental health score after intervention in the counseling group and control group was 14.0 (4.8) and 22.6 (7.0), respectively, which was significantly lower in the intervention group than in control group based on ANCOVA and adjusted baseline values (AMD: -9.8; 95% Confident Interval (CI): -12.5 to -7.1; *P* < 0.001), (Table 2).

**Table 2:** Comparison of mean scores of mental health before and after intervention between groups

Variable	Counseling (*n* = 34) Mean (SD^a^)	Control (*n* = 32) Mean (SD^a^)	MD (CI 95%)^b^	*P*-Value
**Total mental health score** (Range score: 0 to 84)				
Before intervention	19.8 (2.4)	18.6 (2.6)	-1.2 (-2.4 to -0.01)	0.048^c^
After intervention	14.0 (4.8)	22.6 (7.0)	-9.8 (-12.5 to -7.1)	< 0.001^d^

After intervention, the number of participants in the counseling group was 32 and in the control group was 30

^a^Standard Deviation

^b^Mean Difference (95% Confidence Interval)

^c^Independent t-test

^d^ANCOVA

The mean (SD) of the total postpartum bonding score before the intervention was 18.7 (9.1) in the counseling group and 14.9 (9.6) in the control group. There was no statistically significant difference between the two groups before intervention based on the independent t-test (*P* = 0.098). The mean (SD) of the total postpartum bonding score after the intervention was 8 (3.1) and 17.6 (10.5) in the counseling and control groups, respectively, which was significantly lower in the intervention group than in control group based on ANCOVA and adjusted baseline values (AMD: -10.0; 95% CI: -0.6 to 13.9; *P* < 0.001) (Table 3).

**Table 3:** Comparison of mean scores of postpartum bonding before and after intervention between groups

Variable	Counseling (*n* = 34) Mean (SD^a^)	Control (*n* = 32) Mean (SD^a^)	MD (95% CI)^b^	*P*-Value
**Postpartum Bonding score (Range score: 0 to 125)**				
Before intervention	18.7 (9.1)	14.9 (9.6)	-3.4 (-8.5 to 0.7)	0.098^c^
After intervention	8.0 (3.1)	17.6 (10.5)	-10.0 (-13.9 to -6.0)	< 0.001^d^

After intervention, the number of participants in the counseling group was 32 and in the control group was 30

^a^Standard Deviation

^b^Mean Difference (95% Confidence Interval)

^c^Independent t-test

^d^ANCOVA

The mean (SD) weight of infants before the intervention was 1536.8 (343.0) gram in the counseling group and 1692.5 (483.8) gram in the control group. After the intervention, these weights were 3116.6 (685.0) and 3116.3 (739.4) in the counseling group and in the control group, respectively, suggesting no statistically significant difference between the two groups before (*p* = 0.134) and after (*p* = 0.429) the intervention.

The mean (SD) height of infants before the intervention was 40.1 (3.6) in the counseling group and 41.6 (3.8) in the control group. After the intervention, these values were 48.4 (3.7) and 48.7 (4.0) in the counseling group and in the control group, respectively, suggesting no statistically significant difference between the two groups before (*p* = 0.106) and after (*p* = 0.536) the intervention.

The mean (SD) head circumference of infants before the intervention was 29.1 (2.2) in the counseling group and 29.8 (2.4) in the control group. After the intervention, these values were 34.7 (2.2) and 34.4 (2.6) in the counseling group and in the control group, respectively, suggesting no statistically significant difference between the two groups before (*p* = 0.241) and after (*p* = 0.129) the intervention (Table 4).

**Table 4:** Comparison of mean scores of anthropometric indices of infants before and after intervention between groups

Variable	Counseling (*n* = 34) Mean (SD^a^)	Control (*n* = 32) Mean (SD^a^)	MD (95% CI)^b^	*P*-value
**Weight**				
Before intervention	1536.8 (343)	1692.5 (483.8)	155.7 (-49.5 to 361.0)	0.134^c^
After intervention	3116.6 (685.0)	3116.3 (739.4)	108.7 (-164.7 to 382.1)	0.429^d^
**Height**				
Before intervention	40.1 (3.6)	41.6 (3.8)	1.5 (-0.3 to 3.3)	0.106^c^
After intervention	48.4 (3.7)	48.7 (4.0)	0.466 (-1.0 to 2.0)	0.536^d^
**Head Circumference**				
Before intervention	29.1 (2.2)	29.8 (2.4)	0.7 (-0.5 to 1.8)	0.241^c^
After intervention	34.7 (2.2)	34.4 (2.8)	0.6 (-0.2 to 1.3)	0.129^d^

After intervention, the number of participants in the counseling group was 32 and in the control group was 30

^a^Standard Deviation

^b^Mean Difference (95% Confidence Interval)

^c^Independent t-test

^d^ANCOVA

## Discussion

According to the results of the present study, supportive counseling improved mothers’ mental health and postpartum bonding; however, there was no statistically significant difference between the two groups in terms of the neonatal anthropometric index. Mothers addressed the points such as unfamiliarity with the NICU environment, unawareness on the status of the preterm infant, need for support from family and the health team, need to share experiences, and unpredictable status of the infant as the main experiences they had when their infants were hospitalized at the NICU. Therefore, if the support mothers receive from family and health teams is insufficient, their concerns on the condition of their infants would raise [37]. Mothers of infants hospitalized at NICU feel being more supported once they are communicating with mothers with the same conditions and feel that they have more control over managing their current situation by learning from their peers [8]. In the present study, mothers were also allowed to interact with each other in group sessions. Further, after the mothers expressed their feelings and views of their relatives about premature infants, the wrong views and thoughts were corrected.

In the present study, the mean score of mental health in the intervention group was lower than that in the control group, which indicates a better status of the intervention group as compared with the control one. Confirming the results of the research, in the study of Ghodrati et al. [38], training neonatal care during a 45-60-minute session to mothers of premature infants significantly reduced their anxiety levels in 10 days after delivery (*P* = 0.002). In another study, Valizadeh et al. [39] placed 99 mothers of preterm infants into one of three groups: film, booklet, and control. The mothers completed the State- Trait Anxiety Inventory before entering the neonatal intensive care unit. Then, the participants in the intervention group were introduced to the NICU environment via film or booklet. After entering the NICU, once again, this questionnaire and the Cattell Anxiety Questionnaire were completed by the mothers. In their research, mothers in the intervention group (film or booklet) had less anxiety than the control group (*P* < 0.001). In the present research similar to Valizadeh et al.’s study, to make mothers familiar with NICU, the researcher provided them with information on the neonatal intensive care unit, except that the mothers had entered the NICU prior to provision and had met their infants.

In the study of Karami et al. [40], supportive-educational program accomplishment for 2 to 3 sessions was effective in reducing the stress of mothers of preterm infants. The findings of this research may be in line with their results; however, in the former, mothers received 6 sessions of intervention and their mental status was evaluated via the GHQ. Also, Turan et al. [41], in a clinical trial revealed that 30-minute training to parents of premature infants one week after their newborn hospitalization at NICU, explaining on NICU and their infants’ status, and also answering their questions significantly reduced their stress in 10 days after the intervention. In a further clinical trial, Glavin et al. [26] included 228 mothers with infants born alive in their study to investigate the supportive counseling impact on postpartum depression (64 in the control group and 164 in the intervention one); the intervention group received supportive counseling. The mean scores of postpartum depression before intervention were similar between the two groups (*P* = 0.76), while there was a statistically significant difference between the mean scores of depression at 3 and 6 months postpartum (P < 0.01). The positive impact of supportive counseling on mental health has also been reported in other cases such as women experiencing menopause [42], mothers exposed to preterm labor [43], women experienced abortion [44], and cases experienced emergency cesarean [45]. In the study of Chourasia et al. [46] to investigate the “effect of counseling on stress levels of NICU mothers”, interns (medical trainees) provided mothers with accurate medical information on their neonates and their progress state after talking to the neonatal intensive care unit team. The whole process lasted 30–45 minutes. Comparing the maternal stress before the intervention (6 to 8 days after infant’s hospitalization at the NICU) and after the intervention (48 hours after the intervention) revealed that counseling mothers of such neonates at the NICU was effective in reducing their stress (*P* < 0.05). Interventions such as counseling have a positive effect on the mental health of mothers after childbirth [47]. According to a study by Gamble et al. [48], counseling may reduce mothers’ anxiety within the first 72 hours and during the 4 to 6 weeks after delivery. The results of this study are consistent with those of the present study.

Osman et al. [49] reported that training through a 20 munities supportive film on various postpartum stressors and a 24-hour support hotline to answer mothers’ questions by trained midwives are effective in reducing parental stress (*P* < 0.05). Providing counseling to mothers during their infants’ hospitalization at the NICU and after discharge helps them to better manage their unpleasant emotions such as shock and worry about infant care [50]. Since mothers may also face several difficulties in their infant care after leaving the hospital, which in turn affects mental health [51], it is vital to counsel after the discharge. For this issue, the researcher gave her phone number to the mothers so that they could reach the researcher if needed.

The findings of the present study revealed that the total mean score of postpartum bonding in the eight weeks postpartum was lower in the counseling group compared with the control one. In other words, counseling could improve postpartum bonding in the intervention group. In a clinical trial, Moghaddam Tabrizi et al. [52] divided 330 primiparous mothers randomly into two groups of counseling and control (115 in each group) to investigate the impact of family-based counseling on perceived stress and mother-child bonding. Counseling for 4 to 6 sessions (1 to 6 weeks after delivery) could reduce maternal stress and improve mother-child bonding. The results of this study are in line with the present study; however, in the present study, the support was provided by the researcher and families were not involved. Also, the target population in this study was mothers of premature infants. In another work, Arshadi Bostanabad et al. [53] divided 60 fathers of premature infants (in the second day of hospitalization in neonatal intensive care unit) into two intervention (educational-supportive) and control groups. Mothers’ attachment was measured before the intervention and then the fathers in the intervention group received educational (2 sessions of 60 min) and supportive (1 session) interventions for 2 days. They visited their infants in the NICU and exchanged views with their spouses. Once again, the attachment questionnaire was completed by the mothers in both groups. The results suggest the beneficial effect of educational-supportive interventions on maternal attachment. The advantage of this study over the present study was incorporating the fathers in the intervention. However, as the intervention was for short-term, its long-term impact may not be assured because the attachment is also affected by the mental health of mothers [54]. Such an attachment in the postpartum period may be hampered with the prolonged duration of infant hospitalization.

In the present study, there was no statistically significant difference in terms of neonatal anthropometric index between counseling and control groups. Mard Azad et al. [55] investigated the effect of mother counseling on weight gain in neonates and found that the weight of infants in the counseling group was better than the control group. The findings of these researchers are not consistent with those of the present study. The reasons for this discrepancy may be the high pregnancy age (36 weeks and more versus 28 to 33 weeks and 6 days) and mean weight of infants at birth (2367.56 g vs. 1536.8 g) in the study of Mard Azad et al., who provided better conditions for the infants before study. Also, they selected mothers whose infants had left the hospital. Thus, they were away from NICU stressors. Accordingly, due to relaxation and receiving breastfeeding-specific interventions, they could respond better to counseling. Furthermore, SGA infants have less growth [56]. It is of note that it is not clear whether they considered these infants in their study.

In the present study, the insignificant difference in the anthropometric index of neonates may be due to the high number of SGA infants in the intervention group. Although the difference between the two groups concerning the number of SGA infants was not statistically significant, the number was high in the intervention group according to the Fenton chart. Moreover, Masoumi et al. [57] claimed that breastfeeding counseling to mothers of preterm infants might lead to exclusive breastfeeding and better weight gain in infants one month after birth. Pregnancy age and follow-up period in the mentioned study are different from that of the present one. In the present research, since breastfeeding counseling to mothers is done by NICU staff, the intervention was not performed by the researcher.

### Study strengths and limitations

One of the limitations of the present study was the exclusion of illiterate women. The impossibility for the mothers who were from the other cities other than Tabriz and stayed in the mothers’ room to participate in the research and the impossibility for the neonatal anthropometric index assessment (at birth) by the researcher and its insertion from the file were other limitations of this work. On the other hand, early intervention, use of videos, providing educational material for mothers to meet mothers ‘educational needs as a part of supportive counseling, considering mothers’ leisure time to hold counseling sessions, and holding individual counseling for mothers who were not able to take part in the group sessions were the strengths of this study. Furthermore, the researcher’s phone number had been given to mothers. As a result, the mothers in the group receiving supportive counseling could be in contact with the researcher in cases they needed guidance once left the hospital.

## Conclusions

Based on the results of this study, supportive counseling can improve mental health and postpartum bonding in mothers of preterm infants. Maternal mental health has an important role in the formation of postpartum bonding, cognitive and behavioral development of children, and their mental health. Therefore, early intervention is needed to prevent disorder in mother’s mental health. Based on the results of the present research, this counseling method may be recommended to health care providers to offer to mothers.

## Data Availability

Datasets used and analyzed during this study are available from the corresponding author on reasonable request.

## Abbreviations

NICU: Neonatal intensive care unit
AMD: Adjusted mean difference
PBQ: Postpartum Bonding Questionnaire
GHQ: General Health Questionnaire
ICC: Intra correlation coefficient
ANCOVA: Analysis of covariance
SGA: Small for gestational age
